# Novel Scale for Clinical Identification of Adverse Magnesium/Calcium Imbalances: Applications and Perspectives

**DOI:** 10.3390/nu17233662

**Published:** 2025-11-23

**Authors:** Deanna J. Nelson, Andrea Rosanoff, Bodo von Ehrlich

**Affiliations:** 1 BioLink Life Sciences, Inc., 250 Quade Dr, Cary, NC 27513, USA; 2 CMER Center for Magnesium Education and Research, 13-1255 Malama St, Pahoa, HI 96788, USA; 3 Internal Medicine Private Practice, 87435 Kempten, Germany

**Keywords:** calcium, magnesium, chronic latent deficiency, Mg/Ca ratio, Ca/Mg ratio, diagnostic scale

## Abstract

**Background/Objectives:** Calcium (Ca) and magnesium (Mg) are mutual antagonists that interact in a majority of physiological processes. Thus, maintaining a balance between these two minerals is essential for functional homeostasis. Mg deficiency plays a role in the course and severity of a wide range of chronic health conditions. This knowledge, however, has not translated into active incorporation of serum Mg determination as a reliable diagnostic tool for physiological Mg deficit. **Methods:** Pioneering work by von Ehrlich and Rosanoff has prompted development of a simple algorithm and scale for using serum Ca and Mg determinations to identify subclinical (i.e., “hidden”) physiological Mg deficiencies. This novel serum Mg/Ca–Ca/Mg scale uses determinations of total serum Ca and Mg and their ratios to alert clinicians to avoidable risks associated with chronic latent Mg deficiency. This review applies this scale to 10 published clinical studies reporting the serum (or plasma) ratio between Mg and Ca. **Results:** In all 10 studies, application of the serum Mg/Ca–Ca/Mg scale diagnosed deficit Mg status more reliably than serum Mg alone. **Conclusions:** This review provides both illustrations of the value of the novel scale and support for its consideration in clinical practice and Mg research.

## 1. Introduction

**Figure 1:**
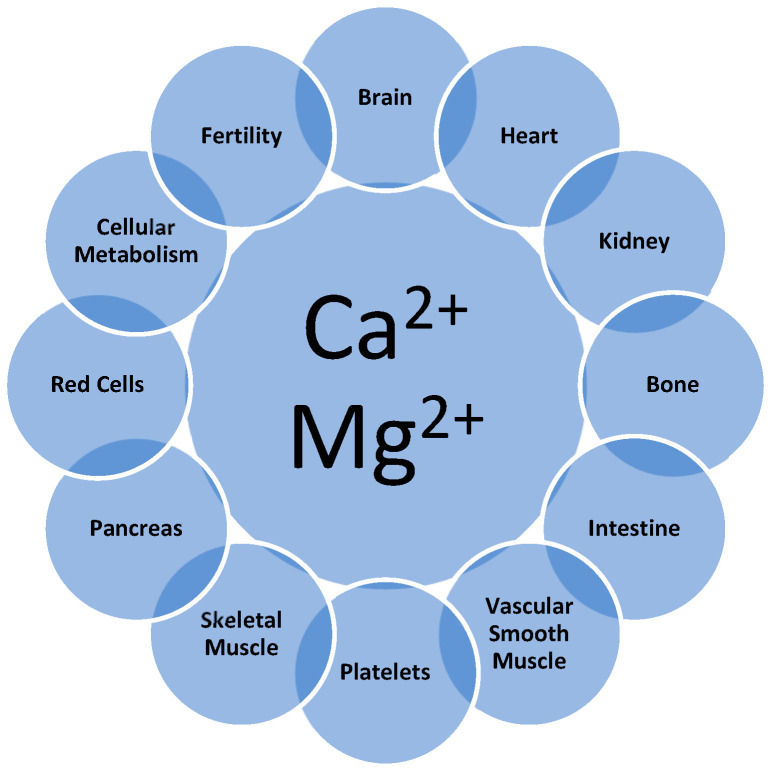
Ca and Mg interactions affecting structure and function in the body. Detailed discussion of these interactions may be found in recent reviews [1,2,3,4].

Calcium (Ca) and magnesium (Mg) are two of the most important minerals in the body. Because Ca and Mg are mutual antagonists that interact in a majority of physiological processes, maintaining a balance between these two minerals is essential [1,2,3,4]. The intricate physiological balance between these two minerals regulates the performance of the intestine, brain, heart, kidney, vascular system, and skeleton (muscle and bone) and significantly impacts the functions of key systems within the body (Figure 1) [5].

Clinicians have known for many decades that Mg deficiency plays a role in the course and severity of a wide range of chronic health conditions (Table 1) [6,7,8,9]. They also recognize that subclinical Mg deficiency may develop within weeks, with physiological repletion requiring several months [10]. Diet is the primary source of mineral replenishment, but dietary quality has changed, as soil mineral depletion and nutrient processing have adversely altered the mineral content of foods [11,12,13,14]. As a result, Mg deficiency is widespread [15,16]. This knowledge, however, has not translated into active incorporation of serum Mg determination as a reliable diagnostic tool for Mg deficiency.

From a clinical perspective, reference ranges for serum Ca are well defined and typically range from 2.2 to 2.7 mmol/L. As a result, serum Ca determinations are widely used diagnostically. In contrast, reference ranges for serum Mg across the world vary more widely and are generally regarded as less useful tools for diagnosis and treatment of Mg deficiency [17,18]. In addition, serum Mg may not always accurately reflect intracellular Mg status. Therefore, considered as a single parameter, serum Mg has limitations as a biomarker of physiological Mg status.

Nonetheless, investigators have called periodically for a useful way to use Mg determinations as tools for diagnosis and treatment. For example, in 1976, Drach evaluated multiple serum and urinary factors in 44 patients with Ca urinary stone disease [19]. Although Drach did not use a scale similar to the novel scale described in Table 2, his treatment (i.e., administration of oral Mg supplements) reflected recognition of the relationship between Ca and Mg and risk of kidney stone formation. In 2004, Liebscher and Liebscher pointed out that Mg deficiency was frequently misdiagnosed, and they recommended that diagnoses be based on critical values of serum Mg (0.85 or 0.9 mmol/L) that were established using statistical foundations [20]. Their report initiated a flurry of similar requests in which multiple approaches for Mg determination in serum, urine, red blood cells, or tissues were named as potential diagnostic tools [8,21]. To date, none of these proposals have been translated into widespread clinical practice.

Pioneering work by von Ehrlich, Rosanoff, and their colleagues has prompted the development of a simple algorithm and scale (the novel serum Mg/Ca–Ca/Mg scale) for using serum Ca and Mg determinations to identify subclinical (i.e., “hidden”) Mg deficiencies, chronic latent deficiencies that are known to exacerbate a wide variety of physiological dysfunctions (Table 1). (See the companion paper by Rosanoff et al., 2025, this issue [22].) The scale was developed using real-world patient data derived from more than three decades of clinical practice; networking with scientific and clinical investigators in the field; presentations and publications for clinical audiences around the world; and global networking with clinical investigators and researchers. Although the scale lacks the statistical support derived from a large, randomized controlled clinical trial, it provides researchers and medical practitioners with an available, inexpensive marker to assess general Mg status, an approach that is open to future research, verification, and proper applicability. It also provides the clinician with a practical way to discuss clinical findings and proposed therapies with the patient.

This novel scale (Table 2) uses determinations of total serum Ca and Mg and their ratios to alert clinicians to avoidable risks associated with chronic latent Mg deficiency. When Mg status is “adequate” according to the scale ratio, no Mg therapy is indicated. However, even “mild Mg depletion” status corresponds to subclinical Mg deficiencies and a recommendation for Mg supplementation in order to present chronic latent Mg deficiency (CLMD). Both “moderate Mg depletion” and “serious Mg depletion” reflect *necessity* for Mg supplementation. Reviews of clinical studies providing support for consideration of the novel scale and illustrations of its value in clinical practice are provided in the sections that follow.

## 2. Materials and Methods

### 2.1. Selection of Studies

**Figure 2:**
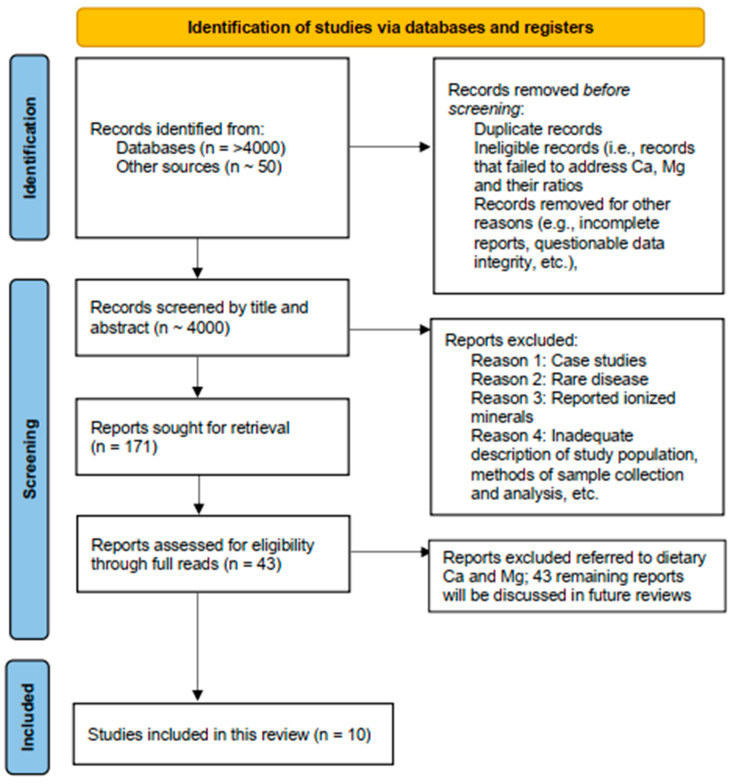
PRISMA flow diagram.

The PRISMA-S search guideline (Figure 2) was used to search scientific and clinical databases, as well as private collections, to identify and analyze >4000 publications [23]. Search terms included *calcium*, *magnesium*, and *calcium magnesium ratio*. Inclusion criteria consisted of articles that focused on determinations of serum Ca and Mg and the ratio between these two minerals (serum Ca/Mg or Mg/Ca), together with their impact on clinical diagnosis and treatment. The exclusion criteria were as follows: (1) articles addressing nutritional status with a focus on dietary Ca and Mg; (2) studies involving fertility, pregnancy and lactation, neonatology, and pediatrics; (3) research focusing on outcomes not related to a balance between the two minerals, including Mendelian analyses; and (4) in vitro or animal studies. Editorials, letters, conference abstracts, and comments were omitted from consideration. Moreover, we scrutinized the reference lists to identify supplementary sources that might prove pertinent. This scoping review is based on works published and incorporated into databases on or before 1 May 2025.

Following the removal of duplicates, both the title and abstract of each of almost 4000 articles were screened for relevance to the topic. Additional articles were obtained from linked research, and reference lists and were screened in the same manner. From these combined sources, a secondary manual screening process led to the identification of a total of 43 studies that were then screened in their entirety for inclusion based on predefined criteria that included the following: (1) type of study (e.g., observational or controlled); (2) class of disease (e.g., chronic or rare); (3) well-characterized subjects (both test and control groups); (4) clinically accepted methodology for sample collection and analysis; and (5) absence of questionable data (e.g., values or units of measure that were outside clinically relevant ranges). Ultimately, 10 articles were selected for inclusion in the present review.

### 2.2. Application of the Novel Serum Mg/Ca–Ca/Mg Scale

Each article was reviewed for the disease or condition being studied, methodology, expression of the serum ratio (see Table 2), mean serum Ca, and mean serum Mg to compare with a standard serum Mg reference range. Our goal was to discern whether application of the novel serum Mg/Ca–Ca/Mg scale and a comparison to published results might add diagnostic value to the reported serum Mg values. For this review, we used 0.7–1.1 mmol/L (1.7–2.7 mg/dL) as the typical serum Mg reference range.

## 3. Results

Findings from application of the novel serum Mg/Ca–Ca/Mg scale to data from 10 published studies are presented next.

### 3.1. Ca/Mg and Metabolic Syndrome

Metabolic syndrome (MetS) is characterized by a cluster of metabolic risk factors such as obesity, dyslipidemia, and hypertension, as well as increased risks of diabetes and cardiovascular disease (CVD) [24]. MetS and its comorbidities pose a significant global health burden. A growing number of studies have reported a significant correlation between low Mg levels and MetS [25,26]. A report by Alsheikh et al., who recently completed a comprehensive cross-sectional study of the association of serum Mg and Ca with MetS, illustrates this relationship [27]. Their study included data collected by the Qatar Biobank (QBB) on 9693 participants aged 20 years or older (mean [±SD] age, 39.9 ± 11.4 years). Participants were randomly selected and categorized into MetS and non-MetS groups using defined criteria for MetS.

The QBB study collected information on sociodemographic factors, lifestyle, and dietary habits through self-administered questionnaires [27]. Nurse interviews were conducted to collect information on participant and family history of health conditions and medication use. All participants underwent a health examination in a single QBB facility at which related clinical laboratory data were collected. Serum levels of Mg and Ca, in addition to recorded metabolic parameters for the study participants, were used in the analyses. Subclinical Mg deficiency was defined as serum Mg levels < 0.85 mmol/L, whereas hypocalcemia was defined as serum Ca levels < 2.1 mmol/L and hypercalcemia as serum Ca levels > 2.6 mmol/L. Serum Ca was corrected for serum albumin levels (*Note:* Ideally, serum Ca would be corrected using albumin levels). Ca/Mg was calculated by dividing the corrected serum concentration of Ca (in mmol/L) by the serum concentration of Mg (in mmol/L). The presence of MetS was deemed as the primary outcome and its components as secondary outcomes. Logistic regression models were run to examine associations between the analytes and metabolic parameters. The models assessing the association of serum Mg and Ca/Mg with MetS were adjusted for age, sex, smoking status, physical activity, diet, and education. Similarly, the model examining serum Ca and MetS was adjusted for the same covariates with the addition of nationality. Secondary logistic regression analyses were conducted to determine the association between each component of MetS with serum Ca, Mg, and Ca/Mg.

MetS was present in >19% of the study population. Subjects in the MetS group had higher triglyceride levels, systolic blood pressure, diastolic blood pressure, fasting plasma glucose levels, and waist circumference as well as lower total cholesterol, high-density lipoprotein (HDL) cholesterol, low-density lipoprotein (LDL) cholesterol, estimated glomerular filtration rate (eGFR), and leisure time physical activity levels than subjects in the non-MetS group. Mean (±SD) serum Mg was higher in the non-MetS group (0.83 ± 0.06 mmol/L) compared to the MetS group (0.81 ± 0.08 mmol/L). In contrast, mean (±SD) serum Ca was higher in the MetS group (2.33 ± 0.09 mmol/L) compared to the non-MetS group (2.30 ± 0.08 mmol/L). Mean (±SD) Ca/Mg was significantly higher in the MetS group (2.92 ± 0.36) than the ratio of means for the non-MetS group (2.77 ± 0.23). Kernel density estimates, which overlapped, showed that the MetS group was mainly distributed in a higher Ca/Mg area than the non-MetS group. In the context of MetS, the data showed a consistent inverse or protective dose–response relationship between serum Mg levels and MetS, and this association remained consistent with most components of MetS. In contrast, serum Ca and Ca/Mg levels demonstrated a positive association with MetS across all quartiles.

In Table 3, we compare clinical interpretation of the data from Alsheikh et al. [27] from a perspective based on a typical reference level of serum Mg (0.7–1.1 mmol/L) and an interpretation using the novel serum Mg/Ca–Ca/Mg scale.

### 3.2. Ca/Mg and Glucose Regulation in Patients with Coronary Artery Disease

Mechanism studies have indicated that Mg and Ca have important biological functions in glucose regulation and are established factors in the risk of coronary artery disease (CAD) [7]. Glycated hemoglobin (HbA1c) is an index of blood glucose control, a significant contributing factor to CAD [7]. In two recent studies, Dong et al. evaluated the associations of Mg and Ca with the hemoglobin glycation index (HGI), abnormal HbA1c, triglyceride-glucose index (TyG), and fasting blood glucose (FBG) in 11,934 Chinese adults with CAD, aged 29–89 years (average age, 61.7 years) [28]. The mediating effects of inflammation were evaluated using high-sensitivity C-reactive protein (hs-CRP) levels. Abnormal HbA1c and abnormal FBG were defined as HbA1c ≥ 6.5% and FBG ≥ 5.6 mmol/L, respectively. Participants were divided into different quartiles according to serum Mg (n = 11,934) and Ca (n = 4606) levels. In multivariable analyses, serum Mg and Ca levels were inversely associated with HGI and TyG. Serum Mg and Mg/Ca were inversely associated with abnormal HbA1c. However, a null association of serum Ca with abnormal HbA1c or Mg/Ca with HGI and TyG was shown. Serum Mg and Mg/Ca were inversely associated with abnormal FBG. In contrast, serum Ca was positively associated with abnormal FBG. Path analysis indicated that there were no mediating effects of hs-CRP on Mg and Mg/Ca–abnormal HbA1c associations. Likewise, path associations indicated that serum Mg and Ca levels had no direct effect on obesity.

Many previous studies have demonstrated that higher circulating Mg concentrations were associated with lower risk of CVD, in part through the protective role that Mg appears to play by regulating glycometabolism [29]. However, emerging evidence from clinical studies provides mixed support for the health benefits of Mg in patients with CVD [30]. In Table 4, we compare clinical interpretation of the Dong et al. [28] data from a perspective based on a typical reference range of serum Mg (0.7–1.1 mmol/L) and an interpretation using the novel serum Mg/Ca–Ca/Mg scale.

### 3.3. Mg/Ca and Ischemic Stroke

Stroke ranks high around the world as a cause of death and long-term disability. Given the current trends toward hypertension, diabetes mellitus, tobacco smoking, and hyperlipidemia, as well as lifestyle factors such as obesity, poor diet/nutrition, and physical inactivity, the incidence of stroke is likely to increase in the next two decades [31]. Acute ischemic stroke accounts for 60% of total stroke; due to the higher mortality and disability rate associated with this condition, the prognosis of acute ischemic stroke remains an important issue. A potential neuroprotective effect of Mg has been presented by several investigators [31,32].

In a recent study, Feng et al. assessed whether serum Mg concentration can predict risk of short-term outcome of acute ischemic stroke [33]. A group of 1493 patients with acute ischemic stroke were recruited from four hospitals in China. Data on demographic characteristics, lifestyle risk factors, history of CVD, admission blood pressure, and other clinical characteristics were collected from all subjects. The short-term outcome was defined as neurological deficiency (National Institutes of Health Stroke Scale [NIHSS] score of <10) or death (NIHSS score of ≥10/death). The Cox proportion hazards regression model was used to evaluate the association between serum Mg concentration and risk of short-term outcome of acute ischemic stroke.

The mean serum Mg concentration in subjects with an NIHSS score of ≥10/death was lower than those with an NIHSS score of <10 (*p* = 0.003). When the highest quartile of serum Mg concentration (≥0.98 mmol/L) was compared with the lowest quartile (<0.83 mmol/L) in an unadjusted model, the risk ratio (RR) was 0.47 (*p* = 0.004), indicating a decreased risk of death in individuals with the highest quartile Mg levels. After adjustment for age, sex, serum Ca concentration, serum potassium concentration, and other covariates, both the third and fourth quartiles of serum Mg concentration were associated with decreased risks of death; the RRs were 0.40 and 0.56 (*p* = 0.027 and 0.001), respectively. Considered as a whole, there was a clear trend toward survival with increasing serum Mg concentrations and higher Mg/Ca molar ratios.

Some previous studies have demonstrated that higher circulating Mg concentrations are associated with lower risk of death from acute ischemic stroke, in part through the protective role that Mg appears to play [34]. However, emerging evidence from clinical studies provides mixed support for the health benefits of Mg in patients with ischemic stroke. In Table 5, we compare clinical interpretation of the Feng et al. [33] data from a perspective based on a typical reference level of serum Mg (0.7–1.1 mmol/L) and an interpretation using the novel serum Mg/Ca–Ca/Mg scale.

### 3.4. Ca/Mg and Cardiac Arrythmias

Cardiologists around the world have acknowledged the significance of Mg deficiency in patients with risk factors for cardiac arrhythmias or manifest rhythm disturbances [35,36]. The use of Mg as a single agent or as an adjunct to other therapeutic actions in the prevention and therapy of cardiac arrhythmias can be both effective and safe [37]. An observational study by Wu et al. illustrates the value of the novel serum Mg/Ca–Ca/Mg scale in assessing the risk of atrial fibrillation (AF) in patients [38].

AF is the most common cardiac arrhythmia and a leading cause of ischemic stroke, heart failure, and cardiac death. Mg exerts antiarrhythmic properties through modulation of myocardial excitability via inhibition of Ca ion entry into the cells. Therefore, an imbalance between these two electrolytes may increase the risk of AF. Wu and colleagues analyzed clinical data from 5792 individuals in an observational study, with incident AF ascertainment in the Atherosclerosis Risk in Communities study [38]. Cox regression models were applied to calculate the hazard ratio (HR) and 95% confidence interval (CI) for AF based on different serum electrolyte levels. Mendelian randomization analyses were performed to examine the causal association. After a median 19.7 years of follow-up, a total of 2551 individuals developed AF. After full adjustment for risk factors such as age, gender, race, hypertension, diabetes mellitus, smoke, drink, body mass index, left ventricular hypertrophy on electrocardiography (ECG), antiarrhythmic medications, plasma creatinine, metabolic equivalent, LDL cholesterol, CRP, and angiotensin-converting enzyme inhibitors, the data indicated that serum electrolyte disorders such as hypokalemia, hypomagnesemia, and hyperphosphatemia were associated with an increased risk of AF and may also serve to be prognostic factors. However, the present study did not support identification of any of the serum electrolytes as causal mediators for AF development.

In Table 6, we present the data provided by Wu et al. in Table 2 of their article [38]. Model 1 adjusted the HR by age, gender, and race. Model 2 adjusted for age, gender, race, hypertension, diabetes mellitus, smoke, drink, body mass index, left ventricular hypertrophy at ECG, antiarrhythmic medications, plasma creatinine, metabolic equivalent, LDL cholesterol, CRP, and angiotensin-converting enzyme inhibitors. Since none of the Ca levels had significance relative to AF, we have used the baseline average serum Ca (9.89 mg/dL [2.47 mmol/L]) to calculate the Ca/Mg molar ratio and compare that value to the novel scale. As can be seen (Table 6), with serum Mg alone as the diagnostic tool, 40% of the patients showed a need for Mg supplementation. When the Ca/Mg molar ratio was calculated and the scale employed, 95% of the patients showed a need for Mg therapy.

### 3.5. Mg/Ca and Chronic Obstructive Pulmonary Disease

Mg is one of the most important factors for regulation of inflammatory response as well as muscle function, and chronic obstructive pulmonary disease (COPD) is a multicomponent disease characterized by abnormal inflammatory response of the lungs with systemic muscle dysfunction. Ruljancic et al. determined concentrations of total Mg (tMg) and ionized Mg (iMg) in plasma and isolated polymorphonuclear (PMN) cells in 46 patients in the stable phase of COPD (past smokers, current smokers, and non-smokers), 24 healthy smokers, and 37 healthy non-smokers [39]. In the plasma and isolated PMN cells of the patients, the ratio of total Ca/total Mg (tCa/tMg) was significantly increased (2.89 [range, 2.15–3.86] and 1.19 [range, 0.07–9.87]) compared to the group of healthy non-smokers (2.65 [range, 2.19–3.44] and 0.67 [range, 0.14–2.40]; *p* < 0.05) and to the group of healthy smokers (2.58 [range, 2.26–3.24] and 0.66 [range, 0.14–2.85]; *p* < 0.05). In the group of patients with COPD, the concentration of tCa was significantly increased in all samples compared to the healthy group of non-smokers and healthy smokers. The results of univariant logistic regression analysis for smoking, concentration of tCa, and tCa/tMg in PMN cells showed high odds ratios (ORs) for COPD status.

In Table 7, we compare clinical interpretation of the Ruljancic et al. [39] data from a perspective based on a typical reference level of serum Mg (0.7–1.1 mmol/L) and an interpretation using the novel serum Mg/Ca–Ca/Mg scale. This study shows that the scale is applicable to plasma ratio of the two cations as well as their serum ratio and that comparing the plasma ratio with the scale can definitively show a statistical difference in Mg status between groups of patients with COPD, healthy smokers, and healthy non-smokers. All three groups of subjects showed a wide range of Mg status using the scale, defining individual needs for Mg therapy in all three groups. The ratio, measured in PMN cells, showed a significant difference between patients with and without COPD, but the scale was not appropriate to define Mg status for this measure.

### 3.6. Ca/Mg and Cystic Fibrosis

Cystic fibrosis (CF) is caused by a genetic defect in epithelial chloride secretion. While pulmonary and gastrointestinal disease remain the most common management challenges, CF also involves significant endocrine disease including diabetes, suboptimal fertility, and abnormal Ca homeostasis with perturbations of the parathyroid hormone (PTH)–vitamin D axis and bone mineral density. Greer et al. [40] evaluated abnormalities of the PTH–vitamin D axis and bone turnover markers in children, adolescents, and adults with CF and included data related to mineral metabolism. The study included a cohort having a different climatic environment, comprising children from age 5 years through adolescents and adults to 56 years, covering a broad disease severity spectrum, and was presented as a reasonably representative sample of the general CF population. The authors found that serum Mg was lower both overall (*p* < 0.0001) and in all CF age groups compared with healthy controls. Low serum Mg in patients with CF was associated with both high and low PTH, low 1,25(OH)_2_D, uncoupled bone turnover, and resistance to vitamin D therapy. Malabsorption and aminoglycoside exposure, common in subjects with CF, are risk factors for Mg depletion. Although the investigators did not see any correlations between serum Mg levels and indices of bone and mineral metabolism, it was speculated that the lower Mg in the CF group in this study might nevertheless have subtle effects on PTH, 1,25(OH)_2_D, and bone turnover and coupling in subjects with CF.

Greer et al. [40] did not use the novel serum Mg/Ca–Ca/Mg scale in their study but their data have been imported into Table 8 to show the potential impact on diagnosis and treatment.

### 3.7. Ca/Mg and Risk of Prostate Cancer

Mg deficiency in Western societies has been linked to some cancers. Dai and colleagues completed two studies of the relationship between Ca/Mg and risk of high-grade prostate cancer [41,42]. In the first study, a biomarker sub-study of the Nashville Men’s Health Study (NMHS), serum Ca and Mg levels were determined in 494 NMHS participants, consisting of 98 high-grade (Gleason score ≥ 7) cancer cases, 100 low-grade cancer cases, 133 prostate intraepithelial neoplasia (PIN) cases, and 163 controls without cancer or PIN at biopsy [41]. Linear and logistic regressions were used to determine associations between serum Ca, serum Mg, and the Ca/Mg weight ratio across controls and case groups while adjusting for potential confounding factors (age, and then waist-to-hip ratio, diabetes treatment, CVD treatment, and race). Dai et al. found that serum Mg levels were significantly lower, whereas Ca/Mg was significantly higher, among high-grade cases vs. controls (*p* = 0.04 and *p* = 0.01, respectively). Elevated Mg was significantly associated with a lower risk of high-grade prostate cancer (OR, 0.26 [95% CI, 0.09–0.85]). Elevated Ca/Mg was also associated with an increased risk of high-grade prostate cancer (OR, 2.81 [95% CI, 1.24–6.36]), adjusted for serum Ca and Mg). In contrast, serum Ca levels were not significantly associated with prostate cancer or PIN. Mg, Ca, or Ca/Mg levels were not associated with low-grade cancer, PIN, prostate-specific antigen levels, prostate volume, or benign prostatic hyperplasia treatment.

In a second study, Dai and colleagues investigated the race-specific link between serum Mg and Ca levels, or dietary Mg intake, and the diagnosis of low-grade and high-grade prostate cancer [42]. The study included 637 prostate cancer cases and 715 biopsy–negative controls (50% were black men). Serum Mg levels and dietary Mg intake were significantly lower in black men compared to white men. However, neither Mg levels nor intake were associated with risk of total prostate cancer or aggressive prostate cancer. Indeed, higher Ca-to-Mg diet intake was significantly protective for high-grade prostate cancer in black men (OR, 0.66 [95% CI, 0.45–0.96]; *p* = 0.03) but not white men (OR, 1.00 [95% CI, 0.79–1.26]; *p* = 0.99). The authors concluded, “In summary, there was a statistically significant difference in Mg intake between black and white men, but the biological impact was unclear” [42].

In Table 9, data from each of the two studies is entered and an interpretation based on the novel serum Mg/Ca–Ca/Mg scale is included.

### 3.8. Ca/Mg and Periodontitis

Mg has important regulatory functions not only in health but also in systemic diseases associated with periodontitis. In these systemic diseases, a chronic low Mg state may be a risk factor for increased severity of periodontitis [43,44]. As part of a cross-sectional Study of Health in Pomerania (Germany) (SHIP-0), Meisel et al. analyzed 5-year follow-up data in 3300 SHIP participants aged 29–62 years (average age, 44–48 years) [45]. The goal was to determine whether baseline Mg levels had a long-term effect on attachment level and number of teeth lost. For 2432 subjects, the authors related the outcome variables of periodontal attachment level and tooth loss to baseline characteristics, especially serum Mg and Ca concentrations, as well as systemic markers of inflammation. (Normal ranges were set at 0.75–1.05 mmol/L and 2.25–2.69 mmol/L for Mg and Ca, respectively.) The progression of periodontitis was associated with the Mg/Ca molar ratio at baseline in a dose-dependent manner. Progression of mean attachment loss was prevented in the upper quartile of Mg/Ca (*p* < 0.001) with antagonistic effects of Mg and Ca, irrespective of inflammatory state. With respect to tooth loss, Mg/Ca exerted dimorphic effects. In inflammatory states as indicated by high C-reactive protein (>3 mg/L), tooth loss was prevented in subjects with high Mg/Ca (incidence rate ratio, 0.60 [95% CI, 0.45–0.80]; *p* = 0.001), but a contrary insignificant trend was observed in subjects with low C-reactive protein levels (incidence rate ratio, 1.14 [95% CI, 0.97–1.34]; *p* = not significant). Similar results were observed with stratifying the regression on tooth loss by interleukin-6 or fibrinogen threshold. These data suggest an adequate Mg serum level and Mg/Ca balance may prevent progression of detachment and tooth loss, especially in inflammatory states.

In Table 10, we compare clinical interpretation of the Meisel et al. [45] data from a perspective based on a typical reference level of serum Mg (0.75–1.05 mmol/L) and an interpretation using the novel serum Mg/Ca–Ca/Mg scale.

### 3.9. Ca and Mg and Kidney Stones

Identification of risk factors for the development of kidney stones represents one of the earliest published applications of diagnosis and treatment based on serum Ca and serum Mg. Drach evaluated multiple serum and urinary factors in 44 patients with Ca urinary stone disease [19]. His findings confirmed a number of defects that have been described in earlier publications (i.e., elevation of mean serum Ca and uric acid above normal, and depression of mean serum Mg). In a related study, Oreopoulos et al. reported that for most patients, serum Mg/Ca values approached urinary Mg/Ca values that were related to kidney stone formation [46].

The Drach [19] data are summarized in Table 11. Although Drach did not use the novel serum Mg/Ca–Ca/Mg scale described in Table 2 of this review, his treatment (administration of oral Mg supplements) reflected recognition of the relationship between Ca and Mg and risk of stone formation.

### 3.10. Ca/Mg and Sickle Cell Disease

Sickle cell disease (SCD) is a group of inherited blood disorders characterized by mutations in the gene that encodes the hemoglobin subunit β (HBB), resulting in the sickle hemoglobin allele βS (HbS). Low levels of Mg have been associated with sickling, increased polymerization, and vaso-occlusion in sickle cells due to erythrocyte dehydration. Antwi-Boasiako et al. hypothesized that an imbalance of the Ca/Mg molar ratio could lead to clinical complications in SCD and evaluated total serum Mg levels. They computed Ca/Mg in 120 patients with SCD (79 with hemoglobin SS [HbSS] and 41 with hemoglobin SC [HbSC] at steady state) and 48 healthy controls (Table 12) [47]. Serum Mg levels were significantly lower in patients with SCD compared to their healthy counterparts (*p* = 0.002). Mg levels were further reduced in patients with HbSS but were not significantly different from patients with HbSC (*p* = 0.584). Ca/Mg was significantly higher in patients with SCD (*p* = 0.031). Although Ca/Mg was higher in patients with HbSC compared to those with the HbSS genotype, the difference was not significant (*p* = 0.101). The authors [47] concluded:

*Magnesium homeostasis is altered in SCD patients, and Mg levels are lower in HbSS patients. Although serum calcium/magnesium ratio is significantly higher in SCD patients compared with healthy controls, there is no significant difference between patients with HbSS and HbSC genotypes. Taken together, the data suggest Mg supplementation may be required in sickle cell patients.*

(p. 547)

## 4. Discussion

Our analyses are based on 10 peer-reviewed studies, and further validation of the novel serum Mg/Ca–Ca/Mg scale is needed. In the future, we expect to analyze as many as 30 additional studies that we identified in our search group (Figure 2) to expand our knowledge of the value of this proposed Mg status marker in current research.

In our research, we have become aware of four settings in which the novel serum Mg/Ca–Ca/Mg scale may be of less value. Follow-up studies will be needed to confirm or deny these knowledge-based assumptions and may lead to identification of other settings in which the proposed scale may be useful.

- *Setting 1: Patients with obesity*. Patients consuming high-fat diets will excrete both Ca and Mg in urine, reducing the serum levels in ways not considered in scale development [48]. Serum minerals will also be altered if the patient with obesity has diabetes. These factors indicate the need to assess the proposed scale in this special population.
- *Setting 2: Patients with renal insufficiency/chronic kidney disease (CKD)*. Patients with CKD, particularly those in advanced stages and/or using renal replacement therapy, will have serum Ca and Mg levels that have been modified by physiological adjustments that attempt to compensate for mineral imbalances, use of phosphate binders that alter mineral balances, and use of other therapies and therapeutics that alter mineral balances [49,50]. Because of these factors, the scale’s utility may be lower in patients with renal insufficiency/CKD and needs to be specifically studied in this special population.
- *Setting 3: Pregnant women and new mothers.* Data concerning the changes in serum Ca and Mg during pregnancy and lactation are sparse. Pregnant women and new mothers experience Ca and Mg mobilization from the maternal skeleton throughout pregnancy and during lactation [51,52]. Mineral mobilization may alter Ca/Mg in this special population, requiring specific testing of the reliability of the scale.
- *Setting 4: Patients who have both low serum Mg and low serum Ca but sufficient Mg/Ca balance.* Under these conditions, the patient’s body may adapt to the lower physiological concentrations of the two minerals. Nonetheless, if the patient is experiencing physiological stress (e.g., rapid growth, recovery from a bone fracture, or pregnancy), supplementation of both minerals may prove beneficial [53]. The scale needs to be specifically tested in these special populations to ensure its reliability.

## 5. Conclusions

In this review, we applied the novel serum Mg/Ca–Ca/Mg scale to 10 statistically analyzed clinical studies in order to show its potential value in identification of chronic latent physiological Mg deficiency in a wide variety of diseases pertinent to clinical settings.

This novel scale was developed via individual case studies in medical practice and is most appropriately applied to the diagnosis and treatment of a single patient (Rosanoff et al., 2025, this issue [22]). This review further expands use of the novel scale to group research and statistical analyses. Briefly summarized, use of serum or plasma Mg/Ca (or Ca/Mg) and the novel scale in all 10 of these examples showed strong potential to prevent missed diagnoses of Mg deficiency, oversights which could have led to inadequate therapy. Taken together, the reports prompt us to echo a conclusion reached by Alsheikh et al. [27]:

*The complex interaction between Ca and Mg at the cellular level necessitates a delicate balance to enhance their effects, and merely having each mineral within its respective normal range fails to capture this dynamic relationship. Ultimately, given the cost-effectiveness and accessibility of serum Ca and Mg measurements in most clinical laboratories along with the Ca: Mg ratio’s value in capturing their combined homeostatic impact, we advocate for incorporating Ca: Mg ratio monitoring into routine clinical practice to support tailored interventions for high risk individuals.*

(p. 8 of 10)

The findings of this review suggest that use of the novel serum Mg/Ca–Ca/Mg scale described herein may prove valuable in achieving this goal in both research and clinical practice.

## Figures and Tables

**Table 1:** Conditions affected by Mg^2+^.

Cardiovascular disease Metabolic syndrome Infectious diseases Respiratory diseases Type 2 diabetes Osteoporosis Hypertension Renal failure Brain volume and function	Liver disorders Gastrointestinal disorders Central nervous system disorders Pancreatitis Sarcopenia Frailty Fertility Pregnancy and lactation Physical decline in aging

**Table 2:** Scale to assess Mg deficit or sufficiency status using serum Ca and serum Mg.

**Values in mg/dL**	**Calculation: $\frac{Ion\left(\frac{mg}{dL}\right)}{Ion\left(\frac{mg}{dL}\right)}$**	
**Mg Status**	**Serum Mg/Ca Weight Ratio**	**Serum Ca/Mg Weight Ratio**
Adequate	≥0.24	≤4.17
Mild Mg depletion	0.218–0.24	4.17–4.59
Moderate Mg depletion	<0.218	>4.59
Serious Mg depletion	≤0.18	≥5.55
**Values in mmol/L or mEq/L**	**Calculation: $\frac{Ion\left(\frac{mmol}{L}\right)}{Ion\left(\frac{mmol}{L}\right)}$ $\mathrm{or}\;\frac{Ion\left(\frac{mEq}{L}\right)}{Ion\left(\frac{mEq}{L}\right)}$**	
**Mg status**	**Serum Mg/Ca**	**Serum Ca/Mg**
Adequate	≥0.4	≤2.5
Mild Mg depletion	0.36–0.4	2.5–2.78
Moderate Mg depletion	<0.36	>2.78
Serious Mg depletion	≤0.3	≥3.33

**Table 3:** Clinical diagnosis using Alsheikh et al. [27] data and a reference range for serum Mg (0.7–1.1 mmol/L).

	MetS (n = 7724)	Non-MetS (n = 1929)
Mean serum Mg, mmol/L *	0.81 ± 0.08	0.83 ± 0.06
Mg status using the typical serum Mg reference range	Adequate	Adequate
Mean serum Ca, mmol/L	2.33 ± 0.09	2.30 ± 0.08
Mean Ca/Mg molar ratio	2.92 ± 0.36	2.77 ± 0.23
Mg status using the novel serum Mg/Ca–Ca/Mg scale	Moderate to serious Mg depletion	Mild Mg depletion
Recommendation based on the novel scale	Increased Mg likely to reduce both acute and long-term risks of MetS	Increased Mg likely to reduce long-term risks of MetS

* Means are presented with SDs.

**Table 4:** Clinical diagnosis using Dong et al. [28] data and a reference range for serum Mg (0.7–1.1 mmol/L).

	Quartile			
	Q1	Q2	Q3	Q4
Mean serum Mg, mmol/L *	0.77 ± 0.04	0.83 ± 0.01	0.88 ± 0.01	0.97 ± 0.12
Mg status using the typical serum Mg reference range	Adequate	Adequate	Adequate	Adequate
Mean serum Ca, mmol/L	2.14 ± 0.08	2.24 ± 0.02	2.31 ± 0.02	2.41 ± 0.02
Mean Mg/Ca molar ratio	0.33 ± 0.02	0.37 ± 0.01	0.39 ± 0.01	0.44 ± 0.07
Mg status using the novel serum Mg/Ca–Ca/Mg scale	Moderate to serious Mg depletion	Mild Mg depletion	Almost adequate	Adequate
Recommendation based on the novel scale	Increased Mg likely to reduce both acute and long-term risks of CAD	Increased Mg likely to reduce long-term risks of CAD	Increased Mg may prevent development of Mg deficiency	No changes to Mg intake recommended

* Means are presented with SDs.

**Table 5:** Clinical diagnosis using Feng et al. [33] data and a reference range for serum Mg (0.7–1.1 mmol/L).

	Quartile			
	Q1	Q2	Q3	Q4
Serum Mg, mmol/L	<0.83	0.83–0.88	0.89–0.97	>0.98
Mg status using the typical reference range	Adequate	Adequate	Adequate	Adequate
Serum Ca, mmol/L *	2.3 (2.2–2.4)	2.3 (2.2–2.4)	2.3 (2.2–2.4)	2.3 (2.2–2.4)
Mean Mg/Ca molar ratio	0.36	0.37	0.40	0.43
Mg status using the novel serum Mg/Ca–Ca/Mg scale	Mild Mg depletion	Mild Mg depletion	Adequate	Adequate
Recommendation based on the novel scale	Increased Mg likely to reduce long-term risks of ischemic stroke	Increased Mg may prevent development of Mg deficiency	No changes recommended	No changes recommended

* Baseline data from Table 1 in Feng et al. [33].

**Table 6:** Clinical diagnosis using Wu et al. [38] data and the novel scale.

**Clinical diagnosis using Wu et al.** [38] **data and a reference range for serum Mg (0.7–1.1 mmol/L [1.7–2.67 mg/dL])**						
**Electrolyte**	**Percentile**	**Range, mg/dL**	***p* Value of the Hazard Ratio**			**Clinical Diagnosis 1**
			**Unadjusted**	**Adjusted Model 1**	**Adjusted Model 2**	
Mg	<5th	0.50–1.39	<0.001	<0.001	<0.001	Moderate to serious Mg deficiency; supplemental Mg indicated
	5 to <20th	1.40–1.49	0.17	<0.001	0.004	
	20 to <40th	1.50–1.59	0.93	0.90	0.80	
	40 to <60th	1.60–1.69	1.00	1.00	1.00	Mg sufficient
	60 to <80th	1.70–1.79	0.96	0.31	0.59	
	80 to <95th	1.80–1.89	0.09	0.01	0.11	
	>95th	1.90–3.10 *	0.90	0.64	0.78	
**Clinical diagnosis based on use of the reference range (Diagnosis 1) or the novel serum Mg/Ca–Ca/Mg scale (Diagnosis 2)**						
**Mineral**	**Percentile**	**Mg, mg/dL (mmol/L)**		**Clinical Diagnosis 1**	**Ca/Mg Molar Ratio**	**Clinical Diagnosis 2**
		**Range**	**Average**			
Mg	<5th	0.50–1.39	0.95 (0.4)	Mg deficient; supplemental Mg indicated	6.18	Serious Mg deficiency; supplemental Mg indicated
	5 to <20th	1.40–1.49	1.45 (0.60)		4.12	
	20 to <40th	1.50–1.59	1.55 (0.64)		3.86	
	40 to <60th	1.60–1.69	1.65 (0.70)	Mg in range; supplemental Mg not supported	3.53	Mg deficient; supplemental Mg indicated
	60 to <80th	1.70–1.79	1.75 (0.72)		3.43	
	80 to <95th	1.80–1.89	1.85 (0.76)		3.25	Moderate Mg depletion; supplemental Mg indicated
	>95th	1.90–3.10 *	2.50 (1.04)		2.38	Mg sufficient
Ca			9.89 (2.47)		—	

* Very high levels of Mg suggest risk of hypermagnesemia.

**Table 7:** Clinical diagnosis using Ruljancic et al. [39] data and a reference range for serum Mg (0.7–1.1 mmol/L).

	Patients with COPD	Healthy Smokers	Healthy Non-Smokers
Serum Mg, mmol/L *	0.85 (0.83) [0.57–1.03]	0.85 (0.87) [0.75–1.07]	0.88 (0.86) [0.64–1.03]
Serum Ca, mmol/L *	2.42 (2.42) [2.20–2.64]	2.28 (2.31) [2.10–2.50]	2.31 (2.36) [2.15–3.32]
Mean Ca/Mg molar ratio *	2.89 (2.91) [2.15–3.86]	2.58 (2.67) [2.26–3.24]	2.65 (2.70) [2.19–3.44]
Mg status using the typical serum Mg reference range	Adequate	Adequate	Adequate
Mg status using the novel serum Mg/Ca–Ca/Mg scale	Moderate to serious Mg depletion	Nearly adequate	Nearly adequate
Recommendation based on the novel scale	Increased Mg likely to reduce both acute and long-term risks of COPD	Increased Mg mainly preventive and likely to reduce long-term risks of COPD	Increased Mg mainly preventive and likely to reduce long-term risks of COPD

* Median (mean) [range].

**Table 8:** Clinical diagnosis using Greer et al. [40] data and a reference range for serum Mg (0.7–1.1 mmol/L).

	Control Subjects (n = 142)		Subjects with CF (n = 149)	
	Adolescents (n = 92)	Adults (n = 50)	Adolescents (n = 87)	Adults (n = 62)
Mean serum Mg, mmol/L	0.86 ± 0.05	0.84 ± 0.06	0.80 ± 0.08	0.73 ± 0.08
Mg status using the typical serum Mg reference range	Adequate	Adequate	Adequate	Adequate
Mean serum Ca, mmol/L *	2.42 ± 0.09	2.38 ± 0.08	2.43 ± 0.07	2.34 ± 0.09
Mean Ca/Mg molar ratio	2.81	2.83	3.04	3.21
Mg status using the novel serum Mg/Ca–Ca/Mg scale	Moderate Mg depletion	Moderate Mg depletion	Moderate to serious Mg depletion	Near serious Mg depletion
Recommendation based on the novel scale	Increased Mg likely to reduce both acute and long-term risks of CF	Increased Mg likely to reduce both acute and long-term risks of CF	Increased Mg likely to reduce both acute and long-term risks of CF	Increased Mg likely to reduce both acute and long-term risks of CF

* Means are presented with SDs.

**Table 9:** Clinical diagnosis using data from Dai et al. [41] and Fowke et al. [42] studies and a reference range for serum Mg (1.7–2.67 mg/dL).

**Data from Dai et al. Study** [41]					
**Group**	**n**	Mean Values *		**Ca/Mg, by Weight**	**Mg Status Using the Scale**
		**Mg (ng/mL)**	**Ca (ng/mL)**		
Age adjusted					
Control	163	2.16	9.70	4.52	Mg adequate to mild depletion Mild to moderate depletion
Prostate intraepithelial neoplasia (PIN)	133	2.18	9.73	4.52	
Low-grade cancer	99	2.14	9.66	4.57	
High-grade cancer	98	2.09 ^	9.82 ^^	4.78	Moderate Mg depletion
Fully adjusted *					
Control	163	2.09	9.81	4.75	Moderate Mg depletion
PIN	133	2.09	9.87	4.79	
Low-grade cancer	99	2.07	9.76	4.78	
High-grade cancer	98	2.03 **	9.91	4.96 ***	Moderate to serious Mg depletion
**Data from Fowke et al. Study** [42]					
Race					
Black	1322	2.3	9.8	4.3	Mild Mg depletion to adequate
White		2.4	9.7	4.1	

* Adjusted for age (continuous), treatment for diabetes (yes or no), treatment for CVD (yes or no), waist-to-hip ratio (categorized at quartiles), and race (white or non-white). The authors report serum Mg and Ca in ng/mL units, but the values reported suggest the units are really mg/dL. If so, the mean serum Mg values in all these groups denote “normomagnesemia,” whereas the serum Ca/Mg weight ratios of each group show adequate for age-adjusted control but mild to moderate Mg depletion for all others. ** High-grade cancer vs. negative controls (*p* = 0.04), vs. PIN (*p* = 0.03), or vs. low-grade cancer (*p* = 0.13). *** High-grade cancer vs. negative controls (*p* = 0.01), vs. PIN (*p* = 0.05), or vs. low-grade cancer (*p* = 0.05). No other differences were significant at *p* < 0.05. Additional adjustment for family history did not change these results. ^ High-grade cancer vs. negative controls (*p* = 0.01), vs. PIN (*p* < 0.01), or vs. low-grade cancer (*p* = 0.09). ^^ High-grade cancer vs. negative controls (*p* < 0.01), vs. PIN (*p* < 0.01), or vs. low-grade cancer (*p* = 0.03).

**Table 10:** Clinical diagnosis using Meisel et al. [45] data and a reference range for serum Mg (0.75–1.05 mmol/L).

	Quartile		
	Q1	Q2–Q3	Q4
Mean serum Mg, mmol/L *	0.70 ± 0.04	0.77 ± 0.04	0.88 ± 0.09
Mg status using the typical serum Mg reference range	Nearly adequate	Adequate	Adequate
Mean serum Ca, mmol/L *	2.48 ± 0.10	2. 41 ± 0.10	2.34 ± 0.11
Mean Mg/Ca molar ratio *	0.28 ± 0.01	0.32 ± 0.01	0.38 ± 0.04
Recommendations based on the novel serum Mg/Ca–Ca/Mg scale	Serious Mg depletion; increased Mg warranted	Moderate to serious Mg depletion; increased Mg warranted	Nearly adequate Mg status; maintain with diet and supplements, if needed

* Means are presented with SDs.

**Table 11:** Clinical diagnosis using data from Drach [19] and a reference range for serum Mg (1.7–2.67 mg/dL).

	Normal Range	Patient Value
Mean serum Mg, mEq/L	2.0	1.76
Mg status using the typical serum Mg reference range	Adequate	Adequate
Mean serum Ca, mg/dL	9.55	9.81
Mean Mg/Ca weight ratio	0.25	0.217
Recommendation based on the novel serum Mg/Ca–Ca/Mg scale	Adequate	Moderate Mg depletion; increased Mg likely to reduce both acute and long-term risks of stones

**Table 12:** Clinical diagnosis using data from Antwi-Boasiako et al. [47] and a reference range for serum Mg (0.7–1.1 mmol/L).

**Electrolyte**	**Healthy Subjects** **(n = 48)**	**Patients with Sickle Cell Disease (SCD)** **(n = 120)**		**Clinical Diagnosis Using Serum Mg Reference Range**	**Clinical Diagnosis Using Novel Scale**
Mean Mg, mmol/L *	0.90 ± 0.11	0.80 ± 0.24		Both groups Mg sufficient	Healthy subjects: Mg adequate Patients with SCD: mild to moderate Mg deficit; initiate or continue Mg supplementation
Mean Ca, mmol/L	2.28 ± 0.53	2.11 ± 0.38			
Mean Ca/Mg molar ratio	2.54 ± 0.89	2.80 ± 0.72			
**Electrolyte**	**Healthy subjects** **(n = 48)**	**Patients with SCD (by subtype)**		**Clinical diagnosis using serum Mg reference range**	**Clinical diagnosis using novel scale**
		**HbSS** **(n = 79)**	**HbSC** **(n = 41)**		
Mean Mg, mmol/L	0.90 ± 0.11	0.79 ± 0.25	0.82 ± 0.21	All 3 groups Mg sufficient	Patients with SCD: mild to moderate Mg deficit; initiate or continue Mg supplementation
Mean Ca, mmol/L	2.28 ± 0.53	2.07 ± 0.39	2.17 ± 0.36		
Mean Ca/Mg molar ratio	2.54 ± 0.89	2.79 ± 0.71	2.82 ± 0.76		

* Means are presented with SDs.

## Data Availability

No new data were created or analyzed in this study; data sharing is not applicable to this article.

## Abbreviations

The following abbreviations are used in this manuscript:

AF	atrial fibrillation
Ca	calcium
CAD	coronary artery disease
CF	cystic fibrosis
CI	confidence interval
CLMD	chronic latent Mg deficiency
COPD	chronic obstructive pulmonary disease
CVD	cardiovascular disease
ECG	electrocardiography
eGFR	estimated glomerular filtration rate
FBG	fasting blood glucose
HbA1c	glycated hemoglobin
HBB	hemoglobin subunit β
HbS	hemoglobin allele βS
HbSC	hemoglobin SC
HbSS	hemoglobin SS
HDL	high-density lipoprotein
HGI	hemoglobin glycation index
HR	hazard ratio
hs-CRP	high-sensitivity C-reactive protein
iMg	ionized Mg
LDL	low-density lipoprotein
MetS	metabolic syndrome
Mg	magnesium
NIHSS	National Institutes of Health Stroke Scale
NMHS	Nashville Men’s Health Study
OR	odds ratio
PIN	prostate intraepithelial neoplasia
PMN	polymorphonuclear
PTH	parathyroid hormone
QBB	Qatar Biobank
RCT	randomized controlled (clinical) trial
RR	risk ratio
SCD	sickle cell disease
SHIP-0	Study of Health in Pomerania (Germany)
tCa	total Ca
tMg	total Mg
TyG	triglyceride-glucose index
